# The power of podcasts: Exploring the endless possibilities of audio education and information in medicine, healthcare epidemiology, and antimicrobial stewardship

**DOI:** 10.1017/ash.2023.178

**Published:** 2023-06-05

**Authors:** Martin A. Kiernan, Brett G. Mitchell, Philip L. Russo

**Affiliations:** 1 Richard Wells Research Centre, University of West London, London, United Kingdom; 2 School of Nursing, Avondale University, Lake Macquarie, New South Wales, Australia; 3 School of Nursing and Midwifery, Monash University, Melbourne, Victoria, Australia; 4 Central Coast Local Health District, Gosford Hospital, Gosford, New South Wales, Australia; 5 Cabrini Research, Cabrini Health, Victoria, Australia

The use of podcasts as a tool for education and professional development has increased in popularity in recent years, and healthcare workers are no exception. With the ever-evolving nature of healthcare and the need for continuous learning, healthcare professionals are turning to podcasts as a convenient and flexible way to stay informed and current. Here, we explore the use of podcasts by healthcare workers, with a focus on their effectiveness in improving knowledge, skills, and practice. Through an unstructured review of the literature, we examined the benefits and challenges of using podcasts in healthcare education in our field, as well as the factors that influence their use. We have also highlighted the potential for podcasts to support interprofessional collaboration and knowledge-sharing among healthcare teams and the role they can play in enhancing patient outcomes.

Podcasts are now well established as a medium through which a variety of audio information can be distributed. The origins of podcasting begin in 2000, when an MP3 player manufacturer MyAudioGo.com began to allow people to upload news stories to its servers and then to be downloaded onto the MP3 players of other users.^1^ Although the company failed within a year, later that year it was suggested that RSS feeds could be used to automatically alert users to a new file being uploaded. The introduction of the first iPod in 2001 then alerted people to the possibility of carrying music with them and by 2003, the first podcasts (as we recognize them today) began to appear in the style of radio shows. Since then, podcasts have become a popular and accessible form of communication to hundreds of millions of listeners. In the United States alone, >100 million people access this form of media.^2^ Podcasts may focus on news, information giving, education, comedy, sports discussion. They are relatively easy to create, free to edit with open-source software such as Audacity (https://www.audacityteam.org/), and economical or even free to host on the Internet. These factors enable a focus on small niches in science and healthcare that may struggle to find a platform in mainstream media. The opportunity to create a new outlet for dissemination, education, and discussion has been taken up by journals, professional societies, and interested individuals, all of whom have created content over the past few years (Table 1). An example of infection control podcasts listed can be readily found in locations like Apple Podcasts, Spotify, Stitcher, Audible, and Google podcast. They are also used as a method of communicating science to members of the public in an accessible form.^3^

**Table 1.** Example*s* of Infection Healthcare Epidemiology– and Antimicrobial Stewardship–Related Podcasts

Podcast	Organizational Affiliation	New Episodes	Year Commenced
Editors in Conversation	American Society for Microbiology	Biweekly	2020
Infectious Disease Puscast	American Society for Microbiology	Biweekly	2022
Meet the Microbiologist	American Society for Microbiology	Monthly	2008
This Week in Microbiology	American Society for Microbiology	Biweekly	2011
This Week in Parasitology	American Society for Microbiology	Biweekly	2009
This Week in Virology	American Society for Microbiology	Weekly	2008
5 Second Rule	Association for Professionals in Infection Control	Monthly	2019
Emerging Infectious Diseases	CDC	Weekly	2007
MMWR Weekly Report	CDC	Weekly	2020
Contagious Conversations	CDC Foundation	Monthly	2019
ECDC: On Air	European Centre for Disease Control	Periodic	2021
Infection Prevention in Conversation	Healthcare Infection Society	Periodic	2022
Let’s Talk ID	Infectious Disease Society of America	Weekly	2012
In Conversation With…	Lancet Infectious Diseases	Periodic	2021
Microbe Talk	Microbiology Society	Monthly	2012
Dirty Drinks	Nebraska ICAP	Monthly	2021
SHEA Podcasts	Society for Healthcare Epidemiology of America	Periodic	2017
The ASHE Podcast	Society for Healthcare Epidemiology of America	Monthly	2022
The ICHE Podcast	Society for Healthcare Epidemiology of America	Monthly	2019
Breakpoints	Society of Infectious Disease Pharmacists	Periodic	2019
CCO Infectious Disease	None	Periodic	2020
Febrile	None	Biweekly	2020
ID:IOTS	None	Biweekly	2021
Infection Control Matters	None	Weekly	2021
Infectious Disease Puscast	None	Biweekly	2022
Let’s Talk Micro	None	Weekly	2021
This Podcast Will Kill You	None	Weekly	2017
Twinning at IPC	None	Periodic	2021

Listening to podcasts has several advantages for healthcare professionals who often have busy schedules, making it difficult for them to attend conferences or courses in person. Informational motives play a role in podcast listening.^4,5^ Podcasts have also been shown to be a useful way of rapidly disseminating information during a pandemic.^6^ The earliest papers on the use of podcasts focused on the utility of podcasts in education as an adjunct to traditional teaching methods, where significant differences in knowledge gained after podcast listening were noted.^7,8^ Podcasts can also be particularly helpful for auditory learners who prefer to listen to information.^4^ A systematic review on the use of social media in graduate medical education has shown some limited evidence of positive learning outcomes of educational podcasts, but a lack of rigor in these studies was an issue.^9^ In a study examining effectiveness of podcast learning that used electroencephalography (EEG) to measure learner attention, podcast listening was preferred to reading in self-reported satisfaction ratings and produced a higher level of learning gain.^10^ Content and style appear important, and although structured podcasts based on curricula may have benefit for learning, narrative podcasts are also an engaging form of obtaining information,^11^ with active aversion to episodes that are simply recorded lectures.^12^

Podcasts offer the convenience of learning on the go. In one study, only 25% of respondents did not undertake any other activity while listening; driving, doing chores, and exercise are popular coactivities.^5^ The length of an educational podcast appears to be important.^13^ In one small study, 15–20 minutes was felt to be the optimal window,^13^ and most participants tended to listen at normal speed rather increased speed.^5^ Healthcare professionals can choose from a wide range of topics and formats, including interviews, panel discussions, and case studies. Podcasts also provide access to experts in a range of areas within healthcare, including clinicians, researchers, and policy makers. This allows professionals to remain current on the latest research and trends in their field. Unlike traditional continuing education options, podcasts are often free or relatively inexpensive, making them an affordable option for healthcare professionals looking to expand their knowledge. They also offer flexibility in terms of content, length, and format.

A number of potential opportunities arise from podcast use, and podcasts may provide a medium for medical researchers to connect with other experts in their field. Many journal-related podcasts, such as those from *Antimicrobial Stewardship & Healthcare Epidemiology* (ASHE) and *Infection Control & Hospital Epidemiology* (ICHE), feature interviews with leading researchers and experts, which can expand the reach of a paper and help researchers expand their professional networks in addition to providing opportunities for collaboration. Professional societies, such as the Society for Healthcare Epidemiology of America (SHEA), Association for Professionals in Infection Control (APIC), the Healthcare Infection Society (HIS), and the American Society for Microbiology (ASM), have all created podcasts to interact and disseminate information to their members. Interested nonaffiliated individuals have also created podcasts. Among others, we created the podcast “Infection Control Matters” in March 2021. To date, we have produced >100 episodes, with >60,000 downloads and listeners in 131 countries. Our experience demonstrates the potential for listenership in a niche of healthcare and the ability of a podcast to break down geographical barriers.

Researchers can use podcasts to find potential collaborators or to learn about research projects that they may be interested in joining. Podcasts can also be a source of inspiration for medical researchers. Hearing about the latest breakthroughs in their field or learning about the challenges faced by other researchers can help researchers to stay motivated and inspired in their own work.

Some disadvantages should also be considered. Unlike live radio, prerecorded podcasts do not allow for immediate interaction or feedback from listeners. This can be a drawback for those who enjoy a live conversation. Listening to a podcast requires an Internet connection, which can be a limitation for people in areas with poor connectivity or limited data plans; however, it is possible to download episodes in bulk on some of the platforms. Audio quality can be an issue, and many podcasts that are locally produced and edited are not of traditional broadcast quality. Podcasts can be time-consuming, with some episodes lasting an hour or more. This can be a challenge for listeners who have limited time or who prefer shorter, more concise content. Although podcasts have become more mainstream in recent years, they are still not as accessible as traditional forms of media such as radio or television. This can be a barrier for people with hearing impairments or those who do not have access to the necessary technology. Another potential drawback is that listeners are limited to those able to understand the language of the podcast. The low barrier to entry for creating a podcast means that vast numbers of shows are available, but not all of them are of high quality, and there is no peer-review process. The listener must rely on their own assessment of the weight they should give to the supplied content. Also, the potential exists for confirmation and selection bias, with the potential for podcasters to give preference to certain topics or for listeners to selectively choose a podcast episode.

In summary, podcasts have been shown to have academic, educational and accessibility benefits. They can be used to remain abreast of current research, and they can stimulate discussion in infection prevention. Although research on podcasts is encouraging, well-designed studies are required to demonstrate the tangible benefits and challenges associated with them. A focus on understanding the optimal structure of podcasts for educational learning in our field is needed. Also, potential benefits or future areas for expansion have not yet been realized, such as the incorporation of podcasts into formal curricula or for making infection prevention information more accessible to a wider audience.
